# Acute fibrinous and organising pneumonia: a case report and review of the literature

**DOI:** 10.1186/1752-1947-3-74

**Published:** 2009-10-12

**Authors:** Argyris Tzouvelekis, Anastasios Koutsopoulos, Anastasia Oikonomou, Marios Froudarakis, Pavlos Zarogoulidis, Paschalis Steiropoulos, Dimitrios Mikroulis, Antonis Antoniades, Demosthenes Bouros

**Affiliations:** 1 Department of Pneumonology, Medical School, Democritus University of Thrace, Alexandroupolis 68100, Greece; 2 Department of Pathology, Medical School, Democritus University of Thrace, Alexandroupolis, Greece; 3 Department of Radiology, Medical School, Democritus University of Thrace, Alexandroupolis, Greece; 4 Department of Cardiothoracic surgery, Medical School, Democritus University of Thrace, Alexandroupolis, Greece; 5 Department of Pneumonology, Serres General Hospital, Serres, Greece

## Abstract

**Introduction:**

Organising pneumonia is a distinct histopathological entity characterized by intra-alveolar buds of granulation tissue, called Masson bodies, which mainly comprise of activated fibroblasts and loose connective tissue. This histopathologic pattern has been described in idiopathic cases, characterizing cryptogenic organising pneumonia as well as in the context of pulmonary infection, drug-induced pneumonitis and following lung transplantation. Although distinct as a clinical and pathological entity, community organising pneumonia may present with atypical clinical and pathological features, such as intra-alveolar fillings of fibrin balls and organising tissue that resembles acute respiratory distress syndrome or diffuse alveolar damage. The latter characteristics constitute a recently described anatomoclinical entity called acute fibrinous and organising pneumonia.

**Case presentation:**

Here, we describe a rare case of acute fibrinous and organising pneumonia, in an otherwise healthy 65-year-old Greek woman who complained of dry cough, fever, weight loss and progressive dyspnoea. She had never been a smoker. Her clinical symptoms showed a rapid deterioration in the two weeks before admission, despite a course of oral antibiotics. After excluding infection and malignancy with routine laboratory tests and flexible bronchoscopy, high resolution computed tomography and video assisted thoracoscopic lung biopsy were performed. Diagnosis was based on radiological features typical of community organising pneumonia coupled with pathologic features characteristic of acute fibrinous and organising pneumonia. The patient was treated with corticosteroids and showed excellent clinical and radiological response three months after treatment initiation.

**Conclusion:**

Acute fibrinous and organising pneumonia is an extremely rare pathologic entity, often misdiagnosed as typical community organising pneumonia. To our knowledge, this is the seventh case of acute fibrinous and organising pneumonia in the literature, with no identifiable cause or association in a female patient, with no underlying lung disease or known exposures and with an unremarkable previous medical history. We highlight the need for careful review of lung biopsies from patients with clinical and radiologic characteristics typical of community organising pneumonia. Although it remains uncertain whether fibrin alters the favourable prognosis and treatment response of community organising pneumonia, it becomes obvious that a thorough pathologic review, apart from establishing the diagnosis of acute fibrinous and organising pneumonia, may predict a more unfavorable outcome therefore alerting the clinician to administer more aggressive and prolonged therapeutic regimens.

## Introduction

The pathological pattern of organising pneumonia associated with intra-alveolar fibrin deposition has been individualised by Beasley et al in 2002 [1] as acute fibrinous and organising pneumonia (AFOP). It was positioned as an under recognized variant of diffuse alveolar damage (DAD), cryptogenic organising pneumonia (COP) and eosinophilic pneumonia (EP). AFOP is an extremely rare, yet not fully accepted as a distinct anatomoclinical, entity that may occur in an idiopathic context or in association with a wide spectrum of clinical conditions, including collagen vascular diseases, adverse drug reactions, occupational or environmental exposures, as well as various infections. AFOP has been reported to present with two clinical profiles of progression, both of them having similar incidences: a fulminant illness often overlapping with acute respiratory distress syndrome (ARDS) and a more subacute clinical profile, with excellent treatment response and prognosis [1], similar to that seen in COP [2]. The radiographic findings of AFOP are almost identical to those seen in COP, consistent with multiple, migratory, patchy, diffuse, alveolar opacities with peripheral and bilateral distribution [1-5]. We have observed crescentic and ring-shaped opacities in COP cases (4), multiple cavitary lesions with hemoptysis [5], and fatal pneumothorax [6].

In this manuscript, we report the seventh case of idiopathic AFOP in a female patient, with no underlying lung disease or known exposures and with an unremarkable previous medical history. The patient presented with a more subacute and mild clinical profile consisting of fever, weight loss and progressive dyspnoea and showed an excellent clinical and radiological response following corticosteroid administration.

## Case presentation

A 65-year-old Greek woman was admitted to the outpatient clinic of our hospital due to dry cough, fever, weight loss of 5 kg and progressive breathlessness for one month. Her clinical symptoms showed a rapid deterioration during the last two weeks before admission, despite a course of oral antibiotics (macrolide and b-lactam). She was a lifetime non-smoker and used to work in an environment with no known exposures to chemicals, fumes, dust and other environmental or occupational allergens. Her previous medical history included osteoporosis and hyperlipidemia, both of them under appropriate medical treatment.

**Figure 1:**
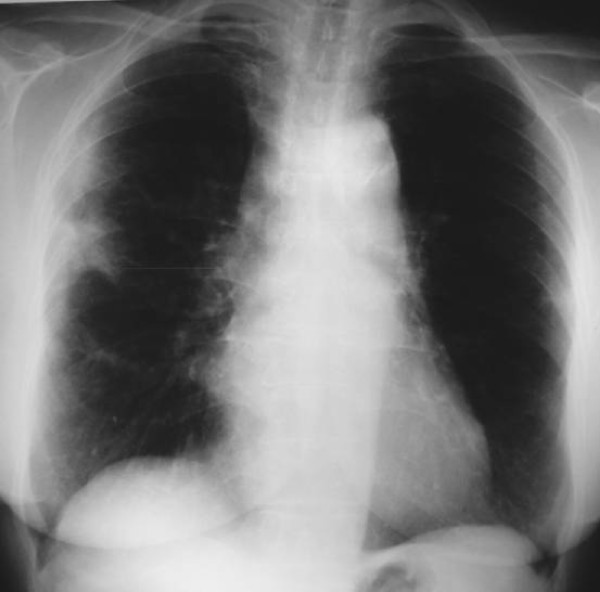
**Posteroanterior chest radiograph at presentation shows patchy areas of consolidation with a predominantly peripheral and subpleural distribution mainly in the right lung**.

**Figure 2:**
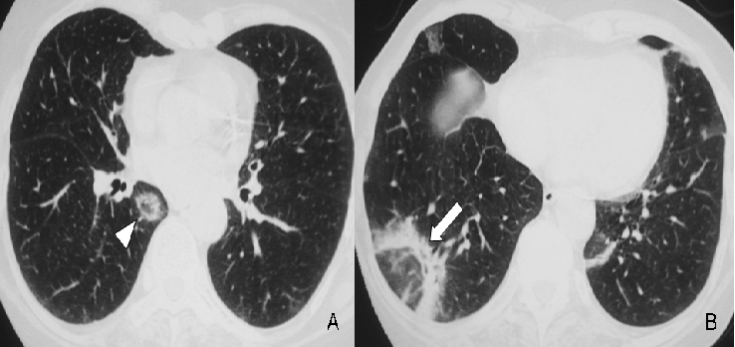
**High-resolution computed tomography (HRCT) at presentation at the level of the lower pulmonary veins (A) and lower lobes (B) at lung windowing shows a ring-like opacity (arrowhead) and subpleural band-like opacity (arrow) with ground-glass in the centre of them consistent with the "reversed halo sign"**.

**Figure 3:**
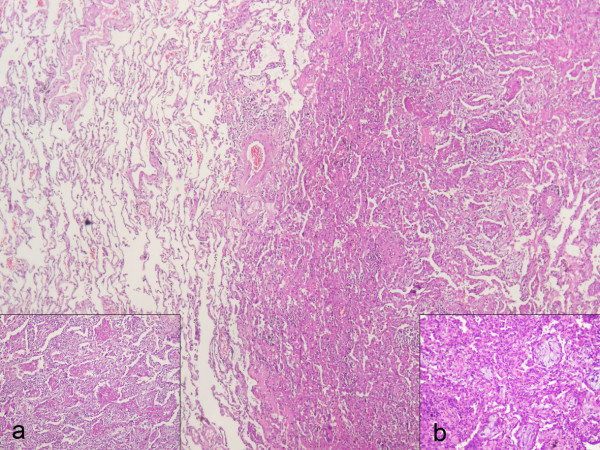
**Histological picture of video-assisted thoracoscopic surgery lung biopsy sample**. Haematoxylin and eosin stain. The specimens from the right lower and middle lung lobes show preservation of the lung architecture with patchy distribution of intra-alveolar fibrin, in the form of fibrin "balls" (insert a), associated with organizing pneumonia, consisting of intraluminal loose connective tissue within the alveolar ducts and bronchioles (insert b). Hyaline membranes, vascular thrombi or eosinophils are absent. The intervening lung parenchyma between the affected areas shows minimal changes, such as a sparse inflammatory infiltrate or minimal interstitial thickening.

On physical examination she was in extremis, had tachypnea (respiratory rate 20/min), and tachycardia (heart rate 100 bpm). She had no hypoxaemia (partial pressure of oxygen 84 mmHg) on arterial blood gas analysis. She had no clubbing, skin lesions, cervical lymphadenopathy or joint swelling. Auscultation of the lungs revealed crepitations with diminished breath sounds over the lower right hemithorax, while inspiratory crackles in the posterior left chest were also noticed. Cardiovascular, abdominal and neurological system examinations were unremarkable. The erythrocyte sedimentation rate was 65 mm.h^-1^. The rest of the physical examination and routine laboratory tests, including white blood cell count and differential, red blood cell count, liver and renal function, and serum C-reactive protein, were normal. Laboratory tests for collagen vascular disease and vasculitis, including ANA, ENA and ANCA antibodies, were also negative. Pulmonary function test parameters were within normal range excluding a slight decrease (60% of predicted value) in carbon monoxide diffusing capacity (DL_CO_). Chest radiograph (Figure 1) followed by high resolution computed tomography (HRCT) of the lung were then performed and showed patchy areas of consolidation with a predominantly peripheral and subpleural distribution mainly in the right lung (Figure 2). The tuberculin skin test was negative. Flexible bronchoscopy followed by bronchoalveolar lavage (BAL) was undertaken and revealed no endobrochial lesion. Bronchial lavage fluid specimens were smear and culture negative for common bacteria and acid-bacilli. In addition, BAL differential cell count revealed a mild lymphocytosis (16%) with a normal CD4/CD8 T cells ratio. In order to exclude malignancy, video-assisted thoracoscopic lung biopsy from multiple lesions of the right lower lobe was undertaken a week after presentation. Extensive pathologic review of lung specimens revealed preservation of the lung architecture with patchy distribution of intra-alveolar fibrin, in the form of fibrin "balls" (Figure 3, insert a), associated with organising pneumonia, consisting of intraluminal loose connective tissue within the alveolar ducts and bronchioles (Figure 3, insert b). The latter observations were consistent with the diagnosis of a rare clinico-pathological entity called AFOP.

**Figure 4:**
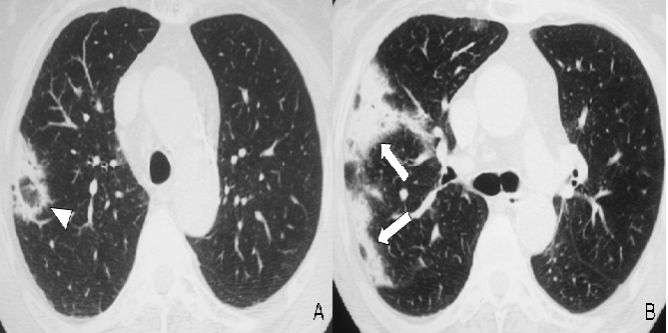
**High-resolution computed tomography (HRCT) two months after discontinuation of treatment at the level of the aortic arch (A) and the carina (B) at lung windowing shows deterioration of the disease with extensive subpleural and peribronchovascular consolidation in the right upper lobe (arrows) and with new ring-like opacities consistent with the "reversed halo sign" (arrowhead)**.

**Figure 5:**
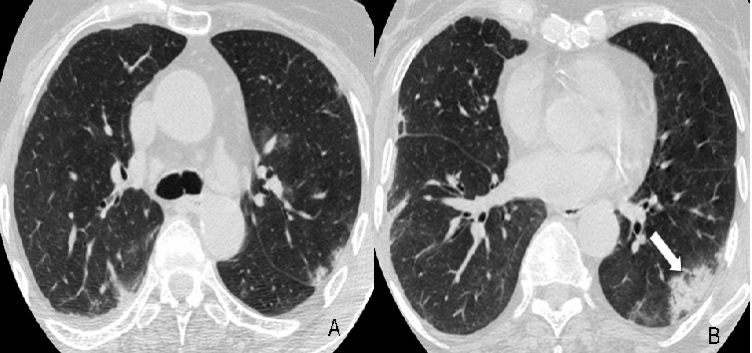
**High-resolution computed tomography (HRCT) after three months of continuous corticosteroid therapy at the level of the carina (A) and lower lobes (B) at lung windowing shows significant improvement with almost complete resolution of most of the previously seen consolidations**. However new small "migratory" subpleural and peribronchovascular opacities are seen in the left lower lobe (arrow) in a previously uninvolved area.

High doses of oral corticosteroid (1 mg/kg of weight) as a monotherapy were then commenced with dramatic symptomatic improvement after 48 hours and near-complete resolution of radiograph infiltrates by the 7th day of treatment. However, she showed poor compliance and discontinued corticosteroid treatment due to prominent side effects including tremor, cushinoid face, growth of facial hair, cataract development in both eyes, mood changes and insomnia. She then presented with relapsing clinical symptoms including fever, progressive dyspnoea and dry cough. HRCT was then undertaken (Figure 4) and demonstrated deterioration of the disease with extensive subpleural and peribronchovascular consolidation in the right upper lobe. She was restarted on oral corticosteroids resulting in rapid clinical and radiological improvement. She remained well and her HRCT (Figure 5) was significantly improved after three months of continuous corticosteroid therapy. The patient is now on a tapering dose.

## Discussion

To the best of our knowledge, this is the seventh case of AFOP in the literature, with no identifiable cause or association in a female patient, with no underlying lung disease or known exposures and with previous medical history almost unremarkable. AFOP is an extremely rare pathologic entity, first described by Beasley et al [1]. While the histologic hallmark of COP is intra-alveolar buds of granulation tissue that share similarities with the fibroblastic foci of usual interstitial pneumonia (UIP) [2], AFOP is also characterized by prominent intra-alveolar fibrin deposition (fibrin balls) resulting in a morphological pattern that may represent an underreported variant of DAD, COP and EP [1-3]. It has been reported that AFOP may occur in an idiopathic context or in association with a wide spectrum of clinical conditions, including collagen vascular diseases, adverse drug reactions, occupational or environmental exposures, as well as various infections. Furthermore, several cases have no identifiable origin or association and consequently are named idiopathic [1-3]. In addition, a case of AFOP in a patient with chronic renal failure being on hemodialysis has also been reported [7].

Retrospective follow-up of these patients revealed two clinical profiles of disease progression, both of them presented with similar incidence: a fulminant illness often overlapping with ARDS, both clinically and pathologically, and a more subacute illness, with favorable treatment response and disease progressiveness [1]. The latter seems to resemble COP in terms of prognosis and recovery following corticosteroid treatment. However, the presence of fibrin in the organising lesions has been associated with less favorable treatment response [2,8]. The radiographic findings of AFOP are almost identical to those seen in COP, consistent with multiple, migratory, patchy, diffuse, alveolar opacities with peripheral and bilateral distribution. In our case, HRCT revealed subpleural and peribronchovascular band-like consolidations with air-bronchograms and no predominant lobe distribution, imaging features typical of COP. However, in addition to that, some of the band-like consolidations formed a peripheral circle engulfing a ground-glass centre, resembling one of the more atypical faces of COP: the "reversed halo sign". HRCT findings of AFOP have been described only in one other case report presenting with a solitary nodule with an air-bronchogram [7], which is also included in the more atypical and rare HRCT appearances of classic COP.

In our case, the clinical presentation of the patient, the almost complete treatment response and the clinical and the radiographic relapse following corticosteroid discontinuation, were typical of COP. There was no evidence of infection, malignancy or any other factors known to be related with organising pneumonia, such as collagen vascular diseases, drug-induced interstitial lung disease or allergic reactions. However, lung biopsy extensively reviewed by an expert pathologist, verified a rather unexpected histologic pattern. Based on the aforementioned data and the mild clinical course of the patient we can conclude that our patient suffered from a more subacute version of the disease. Nevertheless, it is important to stress that the latter seems to be under-diagnosed or misinterpreted as typical COP, as has been demonstrated by recent reports [9,10]. Therefore, we highlight the need of careful reviewing lung biopsies from patients with clinical and radiological characteristics typical of COP. Although, it remains uncertain whether fibrin alters the favourable prognosis and treatment response of COP, it becomes obvious that a thorough pathologic review, apart from establishing the diagnosis of AFOP, may predict a more unfavourable outcome, therefore compelling the clinician to administer more aggressive and prolonged therapeutic regimens.

## Abbreviations

COP: cryptogenic organising pneumonia; AFOP: acute fibrinous and organising pneumonia; DAD: diffuse alveolar damage; EP: eosinophilic pneumonia; ARDS: acute respiratory distress syndrome; DL_CO_: carbon monoxide diffusing capacity; HRCT: high resolution computed tomography; BAL: bronchoalveolar lavage; UIP: usual interstitial pneumonia

## Competing interests

The authors declare that they have no competing interests.

## Authors' contributions

AT performed the physical examination of the patient, analysed clinical and laboratory findings and was a major contributor in writing the manuscript. AK performed the pathologic review of lung specimens, and was a major contributor in writing the manuscript. AO undertook the HRCT and analysed the radiological findings of the disease. MF and PS performed the bronchoscopy and BAL analysis. DM performed the video-assisted thoracoscopic lung biopsy. PZ performed the pulmonary function tests and examined the patient on resubmission. DB and AA analyzed the manuscript for any important intellectual content. All authors read and approved the final manuscript.

## Consent

Written informed consent was obtained from the patient for publication of this case report and accompanying images. A copy of the written consent is available for review by the Editor-in-Chief of this journal.

## References

[B1] Beasley MB Franks TJ Galvin JR Gochuico B Travis WD Acute fibrinous and organizing pneumonia: a histological pattern of lung injury and possible variant of diffuse alveolar damage Arch Pathol Lab Med 2002 126 9 1064 1070 12204055 10.5858/2002-126-1064-AFAOP

[B2] Cordier JF Cryptogenic organising pneumonia Eur Respir J 2006 28 2 422 446 10.1183/09031936.06.00013505 16880372

[B3] Damas C Morais A Moura CS Marques A Acute fibrinous and organizing pneumonia Rev Port Pneumol 2006 12 5 615 620 17117329

[B4] Voloudaki AE Bouros DE Froudarakis ME Datseris GE Apostolaki EG Gourtsoyiannis NC Crescentic and ring-shaped opacities. CT features in two cases of bronchiolitis obliterans organizing pneumonia (BOOP) Acta Radiol 1996 37 6 889 892 10.3109/02841859609175463 8995460

[B5] Kofteridis DP Bouros DE Vamvakas LN Stefanaki KS Voludaki AE Barbounakis EM Emmanouel DS Pneumothorax complicating fatal bronchiolitis obliterans organizing pneumonia Respiration 1999 66 3 266 268 10.1159/000029371 10364745

[B6] Froudarakis M Bouros D Loire R Valasiadou K Tsiftsis D Siafakas NM BOOP presenting with haemoptysis and multiple cavitary nodules Eur Respir J 1995 8 11 1972 1974 10.1183/09031936.95.08111972 8620971

[B7] Kobayashi H Sugimoto C Kanoh S Motoyoshi K Aida S Acute fibrinous and organizing pneumonia: initial presentation as a solitary nodule J Thorac Imaging 2005 20 4 291 293 10.1097/01.rti.0000168600.78213.85 16282908

[B8] Yoshinouchi T Ohtsuki Y Kubo K Shikata Y Clinicopathological study on two types of cryptogenic organizing pneumonitis Respir Med 1995 89 4 271 278 10.1016/0954-6111(95)90087-X 7597266

[B9] Chee CB Da Costa JL Sim CS A female with dry cough, progressive dyspnoea and weight loss Eur Respir J 2005 25 1 206 209 10.1183/09031936.04.00053504 15640343

[B10] Poletti V Casoni GL Cryptogenic organising pneumonia or acute fibrinous and organising pneumonia? Eur Respir J 2005 25 6 1128 10.1183/09031936.05.00004805 15929973

